# Synthetic consensus DNA vaccines against Merkel cell polyomavirus large and small T antigens induce robust polyfunctional T cell responses

**DOI:** 10.3389/fimmu.2026.1840943

**Published:** 2026-09-04

**Authors:** Pratik S. Bhojnagarwala, Elizabeth K. Duperret, Aspen Trautz, David B. Weiner

**Affiliations:** Vaccine and Immunotherapy Center, The Wistar Institute, Philadelphia, PA, United States

**Keywords:** DNA vaccination, large T antigen, Merkel cell carcinoma, Merkel cell polyomavirus (MCPyV), small T antigen

## Abstract

Merkel cell carcinoma (MCC) is an aggressive form of skin cancer with a high mortality rate and limited therapeutic options. Approximately 80% of MCC cases are associated with Merkel cell polyomavirus (MCPyV). The Large T antigen (LTAg) and Small T antigen (STAg) of MCPyV are persistently expressed in tumors and important for viral maintenance and oncogenic transformation of infected cells, making them attractive targets for therapeutic vaccination. Here, we describe the development and preclinical evaluation of DNA vaccines against MCPyV LTAg (LTAgvax) and STAg (STAgvax). Vaccines were designed based on highly conserved regions across MCPyV strains and demonstrated robust expression *in vitro*. Immunization elicited strong antigen specific polyfunctional immune responses, including both CD4+ and CD8+ T cells producing IFNγ, TNFα, and IL2. We observed CD8+ T cells with cytotoxic potential, evidenced by expression of T-bet and CD107a. Importantly, these immune responses were observed in inbred and outbred mouse models, suggesting broad immunogenicity across diverse genetic backgrounds, enhancing the translational relevance of both vaccines. In a minimal residual disease challenge, LTAgvax controlled growth of B16 tumors transduced to express LTAg in mice, improving survival of tumor bearing mice. In a therapeutic challenge, LTAgvax extensively remodeled the tumor microenvironment, with fewer immunosuppressive immune cells and enhanced CD4+ and CD8+ T cell infiltration. The intratumoral CD8+ T cells were more activated and a greater percentage of them had the effector memory phenotype suggesting greater capacity to fight the tumor. This led to improved survival of mice which was further enhanced when mice were co-treated with LTAgvax and anti-PD1 antibody. Together, these findings demonstrate that MCPyV T antigen targeting DNA vaccines induce robust, multifunctional immune responses and control tumor growth. Further development of these vaccines for immunotherapy of MCPyV+ MCC is warranted.

## Introduction

Merkel cell carcinoma (MCC) is a rare but highly aggressive neuroendocrine carcinoma of the skin and is the second largest causes of skin cancer deaths after melanoma (1). MCC disproportionately affects elderly individuals or immunocompromised individuals such as transplant recipients, HIV patients or those with hematologic malignancies (2). Merkel cell polyomavirus (MCPyV) is a major etiological agent in MCC and is associated with approximately 80% of MCC cases (3). For localized MCC tumors, the current standard of care is surgery followed by radiation therapy of the primary tumor (4). For locoregional disease, lymph node dissection combined with radiation therapy is recommended (5). MCPyV- tumors have a high tumor mutation burden (TMB) and are characterized by high infiltration of tumor specific CD4+ and CD8+ T cells, making them good candidates for immune checkpoint inhibitor (ICI) therapy (6, 7). MCPyV+ tumors have a low TMB, but express viral oncoproteins that act as T cell targets making them good candidates for ICI therapy as well. Given this, anti-PD1/PDL1 antibodies are increasingly used for treatment of MCC. These agents have a response rate of 50-70% in the first line setting and ~30% when used as second or later line agents (8–13). Chemotherapy is only recommended in patients with metastatic disease whose tumors are refractory to radiotherapy and ICI (5). Despite these advances the 5-year survival rate for MCC is ~78% for patients with localized disease but as low as 30% for patients with distant metastasis (14), suggesting that there is room for newer therapies for MCC patients.

MCPyV is a major oncogenic driver of MCC tumors. The virus integrates into the host genome, allowing for stable expression of two viral antigens, the large T antigen (LTAg) and small T antigen (STAg). The LTAg binds to tumor suppressor retinoblastoma (Rb), inhibiting its function and allowing for oncogenic transformation of infected cells. STAg is important for viral life-cycle maintenance and is also reported to stabilize LTAg expression (15). Critically, expression of these viral antigens is absent from healthy normal cells, making them excellent targets for therapeutic vaccination.

For therapeutic cancer vaccination, the ideal platform should generate robust CD8+ T cells, be rapid and inexpensive to manufacture and allow for easy delivery (16, 17). Our group has previously developed synthetic DNA (synDNA) vaccines against several cancer antigens including neoantigens and viral antigens such HPV16/HPV18 (E6 and E7) and EBV (Barf1 and EBNA1). These vaccines have demonstrated robust induction of CD4+ and CD8+ T cells in animals and have been effective in controlling lung, ovarian, colon, T lymphoma and melanoma tumors in mouse models (18–24). Clinically, the HPV16/18 E6 and E7 targeting vaccines elicited cellular and humoral immune responses and were effective in elimination of cervical intraepithelial neoplasms (CIN), anal high grade squamous intraepithelial lesions and control of metastatic head and neck cancers (25–29). A phase 1/2 trial of heavily pretreated hepatocellular carcinoma patients treated with a DNA vaccine targeting cancer neoantigens in combination with anti-PD1 antibody demonstrated an overall response rate of 30.3% including 3 complete responses and one surgical complete response, almost doubling the response rate observed with anti-PD1 therapy alone (30). The trial highlights that DNA vaccines can shrink large established tumors and also improve clinical response rates seen with ICI monotherapy.

In this study, we developed and evaluated synDNA vaccines encoding synthetic consensus (synCon) MCPyV LTAg and STAg sequences designed to maximize population coverage and immunogenicity. We assessed antigen expression *in vitro* and characterized vaccine induced cellular and humoral immune responses *in vivo*. Both vaccines led to robust induction of antigen specific CD4+ and CD8+ T cell immune responses as evidenced by expression of IFNγ, TNFα and IL2 by both T cell subsets. The responses were polyfunctional in nature as we saw increased presence of dual and triple cytokine positive CD4+ and CD8+ T cells in mice immunized with either vaccine over those that received empty vector negative control. Importantly, we saw robust immune responses in outbred CD1 mice, highlighting that these vaccines can be effective across diverse MHC types improving their translational relevance. Currently, there are no available mouse models that accurately reflect MCPyV-positive MCC, and MCPyV does not effectively infect mouse cells, which makes modeling viral tumorigenesis in mice challenging. To overcome this, we created a model of B16 (mouse melanoma) cells transduced to overexpress LTAg. In a minimal residual disease model, we observed robust tumor control and significant improvement in survival of tumor bearing mice when vaccinated with LTAgvax compared to empty vector controls. In a therapeutic tumor challenge we observed significant reduction of B16-LTAg tumor size in LTAgvax treated mice which was further enhanced in combination with anti-PD1 antibody therapy.

Mechanistically, LTAgvax significantly remodeled the tumor microenvironment, making it more favorable for CD8+ T cell activity. Tumors treated with LTAgvax had greater infiltration of CD4+ and CD8+ T cells which were more active compared to pVax controls. We also observed greater M1 macrophages and fewer immunosuppressive M2 macrophages and PMN-MDSCs in LTAgvax treated tumors again creating a favorable environment or T cell activity. Finally, these CD8+ T cells were of the effector memory phenotype and had greater expression of tissue resident marker CD69, suggesting that CD8+ T cells in LTAgvax mice were better equipped to kill the tumor and were staying in the tumor longer to continue to kill tumor cells. The data from this study highlight that synDNA vaccines targeting MCPyV LTAg and STAg are highly immunogenic and can control growth of LTAg expressing tumors *in vivo*. These findings support the development of LTAgvax and STAgvax as potential immunotherapy tools for MCPyV+ MCC cancers.

## Results

### Design and expression of MCPyV LTAg and STAg DNA vaccines

**Figure 1:**
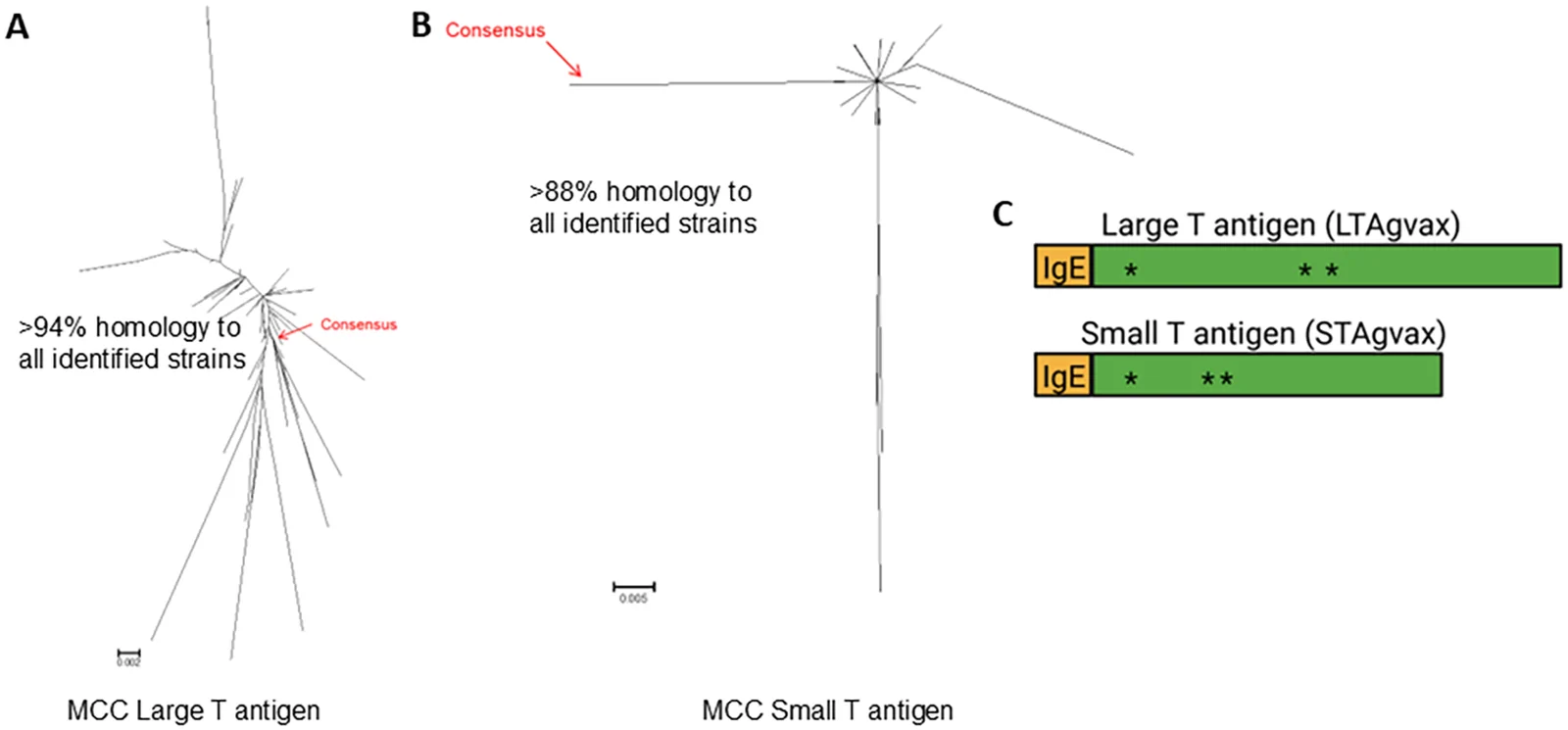
MCC LTAgvax and STAgvax design. Phylogenetic trees for **(A)** LTAgvax and **(B)** STAgvax. Sequences were aligned using Clustal W. Consensus sequence and homology to all identified strains are indicated. **(C)** Representative LTAgvax and STAgvax designs. Asterisks indicate point mutations introduced to abrogate oncogenic properties.

Synthetic Consensus (synCon) sequences for MCPyV LTAg and STAg were generated based on all available viral isolates in the NCBI database. LTAgvax had greater than 94% homology across all identified strains (**Figure 1A**) and the corresponding number for STAgvax was 88% (**Figure 1B**). We introduced point mutations in both vaccines to knock out oncogenicity and viral life cycle maintenance functions. The sequences were then RNA and codon optimized for expression in humans and the human IgE leader sequence was added for improving expression (**Figure 1C**). These optimized sequences were cloned into pVax1 high expression vector and their expression confirmed *in vitro* using western blot. 293T cells transfected with LTAgvax were detected at the right size by the commercial LTAg antibody (**Supplementary Figure 1**) demonstrating expression of the vaccine *in vitro*.

### LTAgvax and STAgvax induce robust cellular immune responses

**Figure 2:**
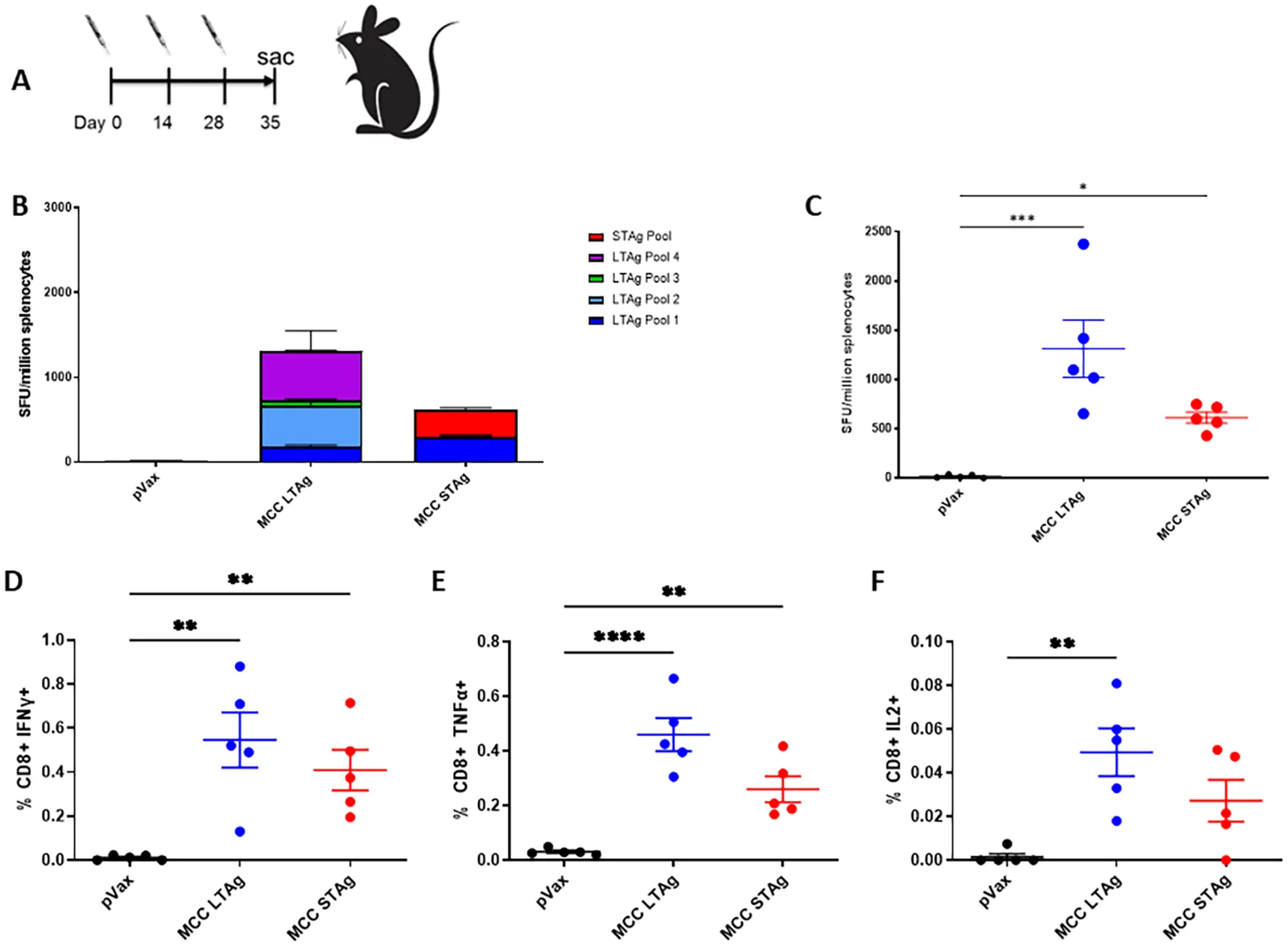
LTAgvax and STAgvax elicit strong immune response in C57bl6 mice. **(A)** Schematic representation of immunization schedule to assess LTAgvax and STAgvax immunogenicity. **(B)** IFNγ spots generated against all peptide pools derived from LTAgvax/STAgvax. **(C)** Sum of all IFNγ spots generated against LTAgvax/STAgvax. **(D-F)** IFNγ, TNFα and IL2 expression on splenic CD8+ T cells from mice immunized with LTAgvax/STAgvax (n= 5 mice/group). ∗p < 0.05; ∗∗p < 0.01; ∗∗∗p < 0.001; ∗∗∗∗p < 0.0001.

To characterize immunogenicity of our vaccines, we immunized C57Bl6 mice 3 times, 2 weeks apart and collected mouse spleens one week after the final dose (**Figure 2A**). IFNγ ELISPOTS demonstrated that all four peptide pools derived from LTAgvax were immunogenic compared to mice injected with empty vector pVax controls. We observed robust induction of IFNγ spots from the LTAgvax against peptide pools derived from LTAg. We also observed immune response against peptide pool 1 of LTAg (**Figure 2B**). This is expected as there is 100% homology between the first 79 amino acids of the two constructs. The total immunogenicity measured by the sum of all IFNγ ELISPOTs also shows that LTAgvax and STAgvax induce robust T cell responses in mice (**Figure 2C**).

To identify immunodominant epitopes from each vaccine, the IFNγ ELISPOT assay was performed using peptide pools the results of which are shown in **Supplementary Figure 2**. Peptides 61–70 was the most immunogenic of all pools tested (**Supplementary Figure 2A**). This pool contains several epitopes which are predicted to have high or medium binding affinity to H2-Kb/H2-Db and are within the top 2 percentile based on predicted immunogenicity of 9-mer epitopes derived from LTAgvax. Pool comprised of peptides 21–30 and 111–120 were respectively the 2^nd^ and 3^rd^ most immunogenic pools. These pools also contain several epitopes that are within the top 2 percentile based on predicted immunogenicity of LTAgvax derived 9-mer epitopes. Similarly, for the LTAgvax the most immunogenic pool (peptides 1-10) contains 6 epitopes with predicted high-medium binding affinity to H2-Kb/H2-Db and are within the top 2 percentile of predicted immunogenic epitopes of 9-mer epitopes derived from STAgvax. These data suggest that immunogenicity elicited by LTAgvax and STAgvax is in line with *in silico* predictions highlighting the strength of our vaccines. The full list of immunodominant epitopes from LTAgvax and STAgvax are listed in **Supplementary Table 1** and **Supplementary Table 2** respectively.

To further characterize the immune response, we performed flow cytometry on these splenocytes after co-culture with peptide pools. LTAgvax and STAgvax induced strong CD8+ T cells as evidenced by increase in expression of IFNγ, TNFα and IL2 on CD8+ (Gating strategy in **Supplementary Figure 3**) T cells in mice immunized with either vaccine (**Figures 2D–F**). LTAgvax also induced a robust CD4+ T cell response as we observed increase in expression of all three cytokines on CD4+ T cells from LTAgvax mice compared to those from negative control mice (**Supplementary Figure 4**). To measure humoral immune response, western blot was performed on 293T cells transduced to express LTAg or STAg and serum from mice immunized with respective construct was used as a primary antibody. Both vaccines induced antibody responses as we observed bands at the appropriate sizes suggesting that the vaccines induce both cellular and humoral immunity. No bands were observed when sera from pVax injected mice was used, when sera from LTAgvax mice was used against STAg transfected cells and vice versa (**Supplementary Figure 5**).

### LTAgvax and STAgvax induce polyfunctional CD8+ T cells with cytotoxic potential

**Figure 3:**
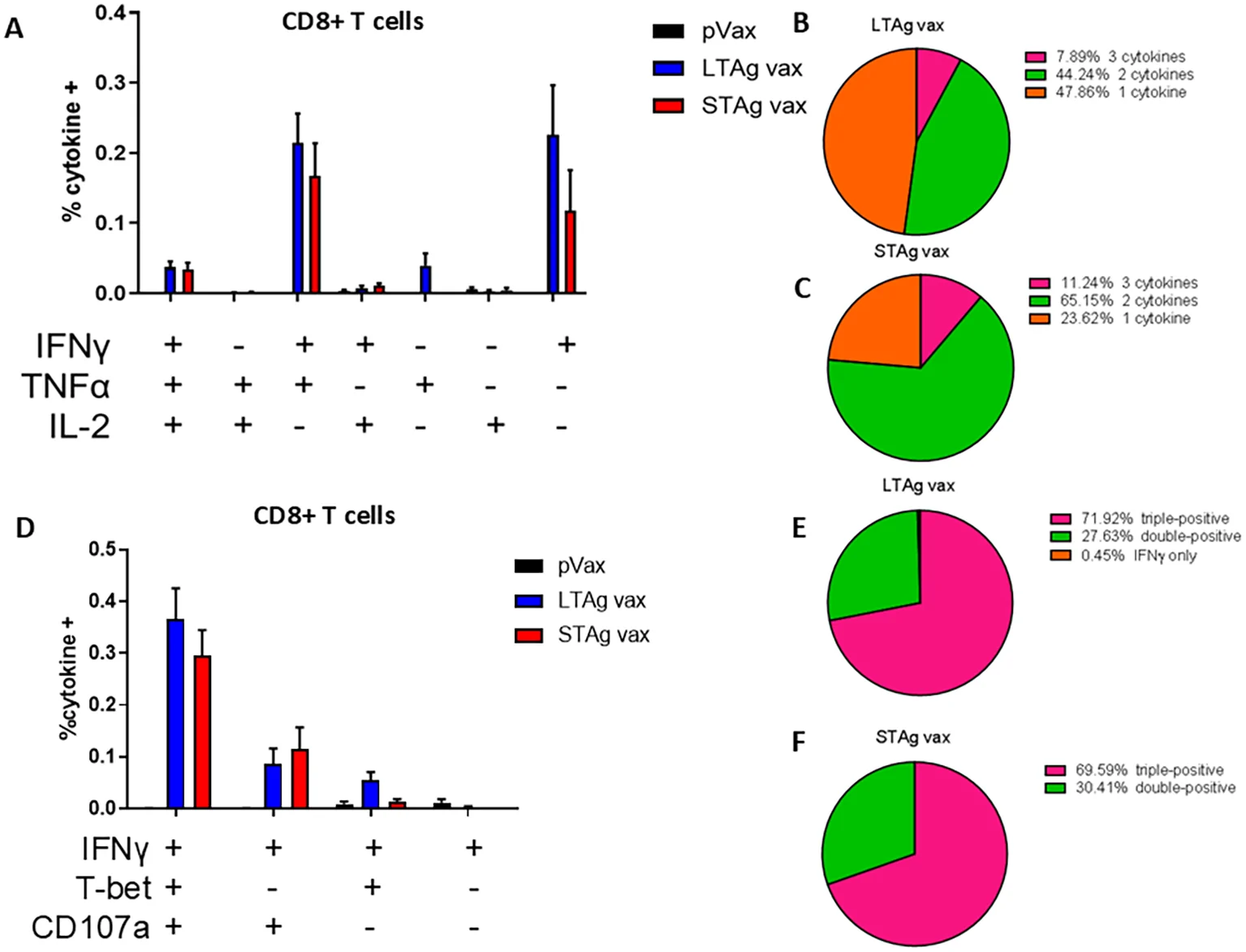
LTAgvax and STAgvax generate polyfunctional CD8+ T cells with cytolytic potential. **(A)** Bar graphs showing CD8+ T cells expressing IFNγ, TNFα and IL2 cytokines after immunization with LTAgvax or STAgvax. **(B)** Pie chart showing percentage of activated CD8+ T cells expressing 3 (pink), 2 (green) or 1 (orange) cytokine after immunization with LTAgvax. **(C)** Pie chart showing percentage of activated CD8+ T cells expressing 3 (pink), 2 (green) or 1 (orange) cytokine after immunization with STAgvax. **(D)** Bar graphs showing CD8+ T cells expressing IFNγ, T-bet and CD107a after immunization with LTAgvax or STAgvax. **(E)** Pie chart showing percentage of activated CD8+ T cells that are triple positive (pink), double positive (green) or IFNγ positive only (orange) after immunization with LTAgvax. **(F)** Pie chart showing percentage of activated CD8+ T cells that are triple positive (pink), double positive (green) or IFNγ positive only (orange) after immunization with STAgvax (n= 5 mice/group).

Boolean gating was performed for deeper characterization of the T cell responses. LTAgvax and STAgvax induced polyfunctional CD8+ T cells as evidenced by presence of IFNγ, TNFα and IL2 triple positive cells as well as double positive cells (**Figure 3A**). ~52% of the CD8+ T cells induced by LTAgvax expressed either 3 or two cytokines (**Figure 3B**). STAgvax induced polyfunctional T cells were greater as ~76% of the induced CD8+ T cells expressed either 2 or 3 cytokines (**Figure 3B**). LTAgvax also induced polyfunctional CD4+ T cells with ~79% of these cells expressing at least 2 cytokines (**Supplementary Figure 5**). Both vaccines also induced CD8+ T cells with cytolytic potential as we observed expression of T-bet and CD107a (**Figure 3D**). Almost 72% of CD8+ T cells induced by LTAgvax were triple positive for IFNγ, CD107a and Tbet and 27.6% were double positive. Less than 0.5% of LTAgvax induced CD8+ T cells were single positive for IFNγ (**Figure 3E**). Almost 70% of STAgvax induced CD8+ T cells were triple positive for IFNγ, CD107a and Tbet while the remaining CD8+ T cells were double positive (**Figure 3F**). These data highlight that both vaccines induced robust polyfunctional T cells expressing multiple cytokines and having high cytolytic potential.

### Immune responses to LTAg and STAg in outbred mice

**Figure 4:**
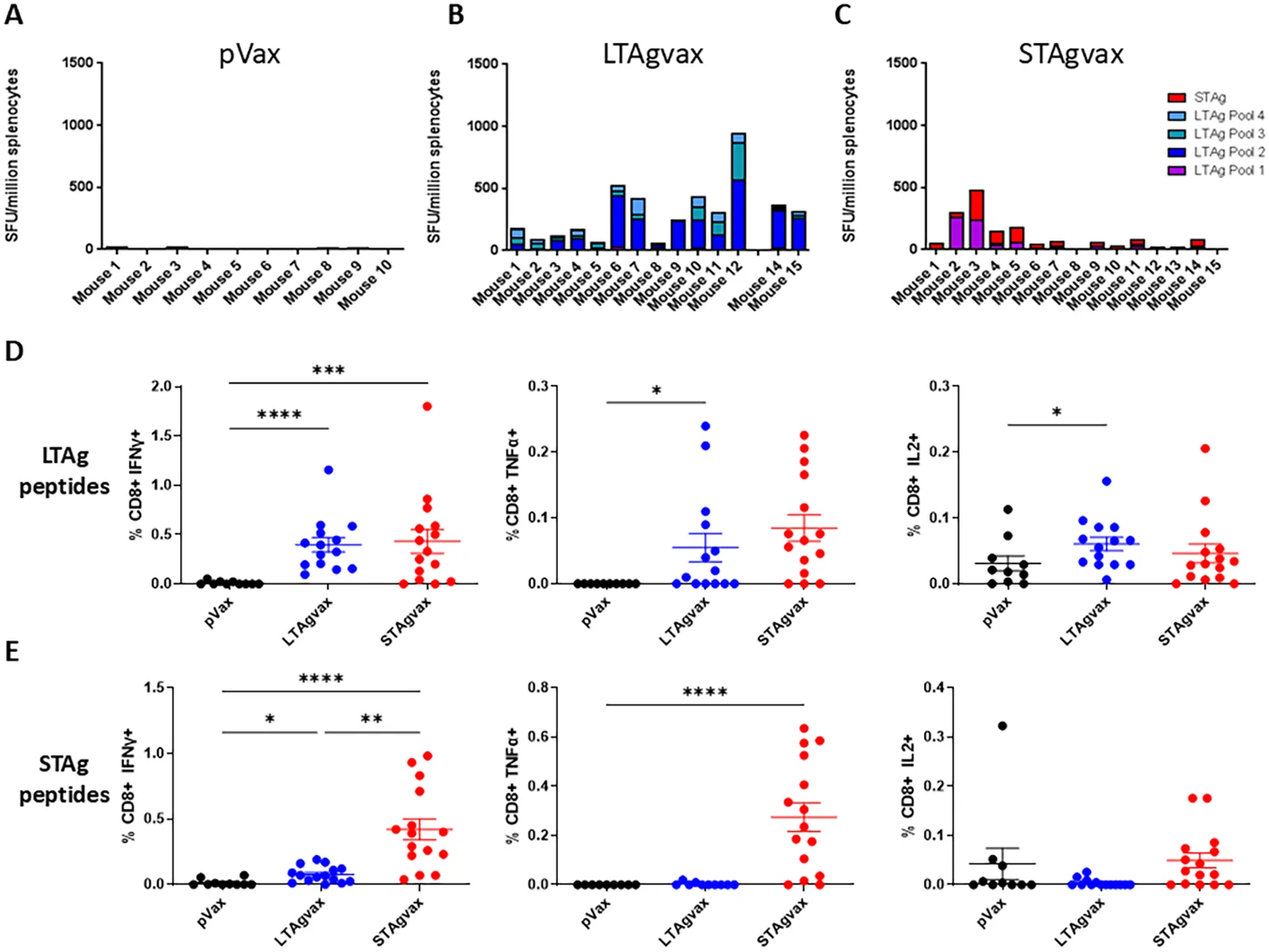
LTAgvax and STAgvax are immunogenic in outbred CD-1 mice. **(A-C)** IFNγ spots observed in CD-1 mice injected with either **(A)** pVax, **(B)** LTAgvax **(C)** STAgvax against the indicated peptide pools. **(D)** CD8+ IFNγ, TNFα and IL2 responses in CD-1 mice after stimulating splenocytes from mice injected with either pVax, LTAgvax or STAgvax with peptides derived from LTAg. **(E)** CD8+ IFNγ, TNFα and IL2 responses in CD-1 mice after stimulating splenocytes from mice injected with either pVax, LTAgvax or STAgvax with peptides derived from STAgvax (n= 10 for pVax and 15 for the vaccinated groups). ∗p < 0.05; ∗∗p < 0.01; ∗∗∗p < 0.001; ∗∗∗∗p < 0.0001.

To test whether LTAgvax and STAgvax would generate immune responses in more genetically diverse mice, we tested immunogenicity in CD-1 mice as described in **Figure 2A**. pVax injected mice showed no spots (**Figure 4A**). 14/15 mice immunized with LTAgvax generated IFNγ spots against at least one LTAg derived peptide pool (**Figure 4B**). 13/15 mice immunized with STAgvax generated IFNγ spots against LTAg peptide pool with 4 mice also generating immune response against Pool 1 derived from LTAg (**Figure 4C**). Deeper analysis of immune responses revealed that with the LTAgvax, 73.4% of the CD-1 mice responded to multiple pools with 46.7% of mice responded to 3 different pools (**Supplementary Figure 6A**).

Flow cytometry analysis revealed LTAgvax and STAgvax generated CD8+ T cell responses as expression of IFNγ, TNFα and IL2 was observed when stimulated with peptides from LTAg (**Figure 4D**) or STAg (**Figure 4E**). Both vaccines also induced CD4+ T cells, and we observed expression of all three cytokines (**Supplementary Figures 7B, C**). These data highlight that both vaccines generate robust T cell responses in genetically diverse CD-1 mice.

### LTAgvax controls tumor growth and improves survival of tumor bearing mice in a minimal residual disease challenge

**Figure 5:**
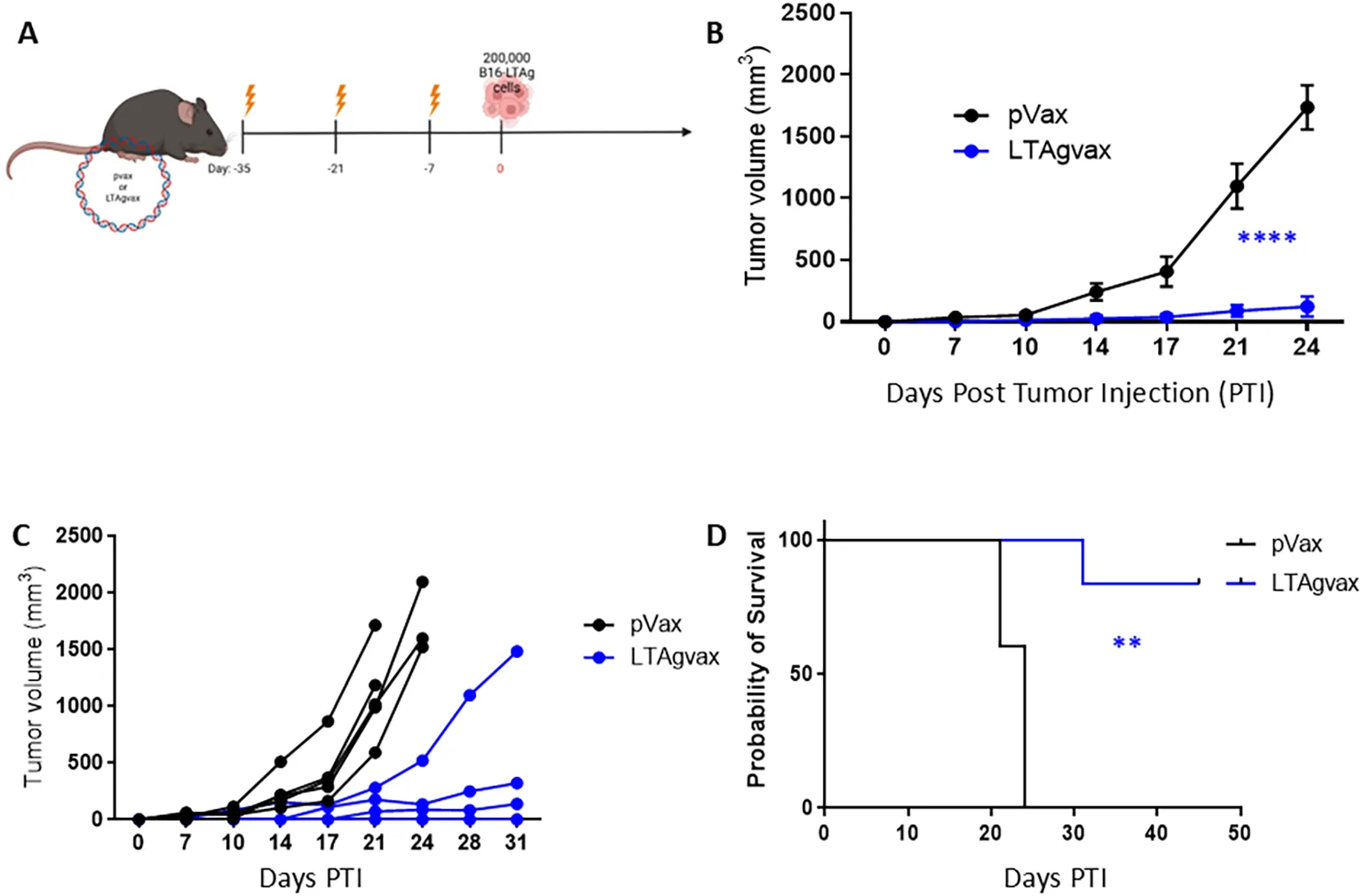
LTAgvax controls growth of B16-LTAg tumors *in vivo*. **(A)** Schematic representation of tumor challenge experiment. **(B)** Mean B16-LTAg tumor size in mice treated with either empty vector pVax control or LTAgvax. **(C)** Individual B16-LTAg tumor size in mice treated with either empty vector pVax control or LTAgvax. **(D)** Survival of B16-LTAg tumor bearing mice, showing improved survival of mice treated with LTAgvax. ∗∗p < 0.01; ∗∗∗∗p < 0.0001.

To test whether LTAgvax can control LTAg expressing tumors, we transduced B16 melanoma cell line with LTAg (B16-LTAg) using retroviral transduction as previously described (22). C57BL6 mice were injected with LTAgvax 3 times at 2 week intervals and one week after final dose, we injected B16-LTAg cells on the right flank of the mice and followed tumor growth and animal survival (**Figure 5A**). LTAgvax slowed down tumor growth and 24 days post tumor injection, LTAgvax treated mice had significantly smaller tumors compared to pVax treated mice (**Figure 5B**). 2/6 LTAgvax treated mice showed slower tumor growth compared to pVax mice and 50% of the mice had a complete response and showed no evidence of tumor growth up to 31 days post tumor injection (**Figure 5C**). Mice receiving pVax had a median survival of 24 days while those receiving LTAgvax did not reach median survival 45 days post tumor challenge leading to a significant improvement in the survival of B16-LTAg tumor bearing mice (**Figure 5D**).

### LTAgvax synergizes with anti-PD1 therapy to improve tumor control in a therapeutic challenge in mice

**Figure 6:**
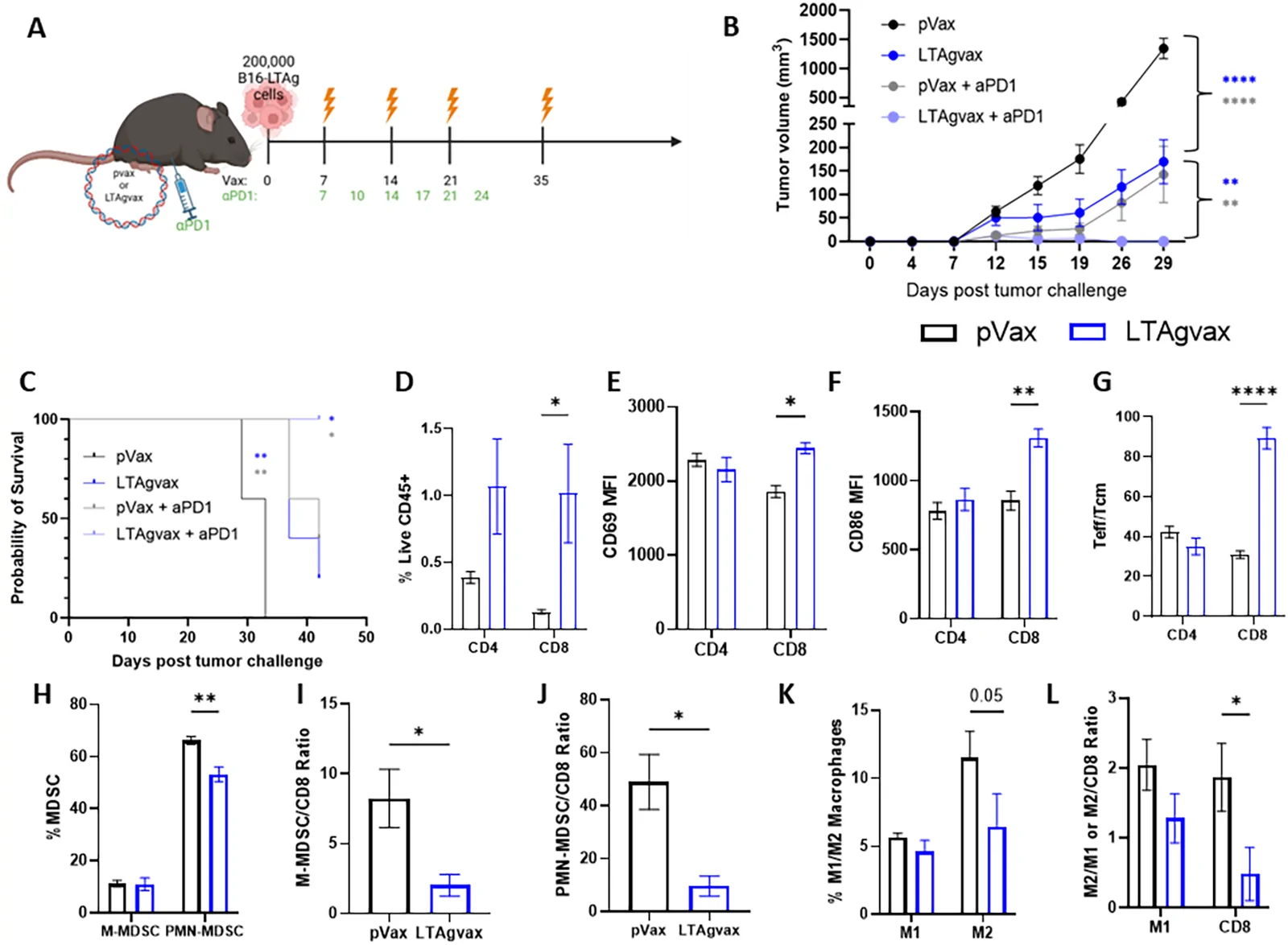
LTAgvax synergizes with anti-PD1 therapy to improve tumor control in a therapeutic challenge in mice. **(A)** Schematic representation of therapeutic tumor challenge. **(B)** Mean B16-LTAg tumor size in mice treated with either pVax, LTAgvax, anti-PD1 antibody alone or both. **(C)** Survival curves of B16-LTAh challenged mice treated with either pVax, LTAgvax, anti-PD1 antibody alone or both. **(D)** percentage of CD4+ and CD8+ T cells infiltrating tumors treated with either pVax (Black) or LTAgvax (Blue). **(E)** CD69 and **(F)** CD86 MFI on CD4+ and CD8+ T cells infiltrating tumors treated with either pVax (Black) or LTAgvax (Blue). **(G)** Ratio of Teff/Tcm CD4+ and CD8+ T cells infiltrating tumors treated with either pVax (Black) or LTAgvax (Blue). **(H)** Percentage of M-MDSC and PMN-MDSC infiltrating tumors treated with either pVax (Black) or LTAgvax (Blue). **(I-J)** M-MDSC/CD8+ T cells and PMN-MDSC/CD8+ T cells ratio within tumors treated with either pVax (Black) or LTAgvax (Blue). **(K)** Percentage of M1 and M2 macrophages in tumors treated with either pVax (Black) or LTAgvax (Blue). **(L)** Ratio of M1 macrophages/CD8+ T cells and M2 macrophages/CD8+ T cells within tumors treated with either pVax (Black) or LTAgvax (Blue). ∗p < 0.05; ∗∗p < 0.01; ∗∗∗∗p < 0.0001.

anti-PD1/PDL1 antibodies have a success rate of 50-70% when used as 1^st^ line MCC therapy (8–12). We wanted to test if this can be improved in combination with our vaccine. A therapeutic tumor challenge was performed where mice were injected with B16-LTAg tumors and one week later, treated with LTAgvax, anti-PD1 antibody or combination of both (**Figure 6A**). Monotherapy with LTAgvax controlled the growth of B16-LTAg tumors including 1/5 mice that showed complete tumor control. anti-PD1 monotherapy also controlled B16-LTAg growth and 2/5 mice demonstrated complete control. We observed strong synergy with LTAgvax was combined with anti-PD1 therapy as 5/5 mice demonstrated complete control (**Figure 6B** and **Supplementary Figure 8A**). Monotherapy with either LTAgvax or anti-PD1 significantly enhanced survival of tumor bearing mice compared to pVax treated mice and dual treatment further enhanced survival of these mice with 100% of the mice being alive 42 days post tumor challenge (**Figure 6C**).

### LTAgvax remodels the tumor microenvironment creating an immune-permissive landscape for greater tumor killing

To better understand the mechanism behind the enhanced tumor control in LTAgvax treated mice, 3 tumors from pVax and LTAgvax treated mice were isolated at the terminal timepoint. These were broken down into a single cell suspension and analyzed for immune infiltration using flow cytometry. Compared to pVax treated tumors, LTAgvax treated tumors had significantly higher CD8+ T cell infiltration and a trend towards greater CD4+ T cell infiltration (**Figure 6D**). Importantly, the CD8+ T cells in LTAgvax treated tumors were significantly more activated, evidenced by significantly higher expression of CD69 and CD86 as well as higher expression of PD1 on their cell surface (**Figure 6E**, 6F and **Supplementary Figure 8B**). Boolean gating on CD8+ T cells from pVax treated tumors demonstrated that 43.85% expressed at least one activation marker, 28.5% expressed two activation markers and 27.65% expressed no activation markers. There were no CD8+ T cells in the pVax treated tumors that expressed three activation markers. The proportion of non-activated tumor infiltrating CD8+ T cells shrunk by more than 50% in LTAgvax treated tumors as only ~11% expressed no activation markers. 55.54% of CD8+ T cells expressed at least one activation marker, 32.07% expressed at least 2 activation markers and 1.36% expressed all three activation markers indicating that CD8+ T cells from LTAgvax treated tumors were more activated compared to those from pVax treated tumors (**Supplementary Figure 8C**).

We also analyzed the T cell memory formation induced by LTAgvax via flow cytometry on tumor infiltrating T cells. We observed a significant increase in the ratio of CD8+ Teff/Tcm cells in the LTAgvax treated tumors (**Figure 6G**). The CD8+ Teff cells from LTAgvax tumors also expressed a higher percentage of CD69 compared to those from pVax tumors suggesting that these are transitioning to a tissue resident phenotype, staying longer in the TME and will continue to kill the tumors for longer (**Supplementary Figure 8D**). We observed a decrease in the CD4+ Teff/naïve T cell ratio and an increase in the CD8+ Teff/naïve T cell ratio in the LTAgvax treated tumors (not reaching statistical significance) suggesting that more CD8+ T cells in the TME of LTAgvax tumors are antigen experienced and primed to continue killing the tumors (**Supplementary Figure 8E**).

Myeloid compartment analysis demonstrated a minor increase in the tumor infiltrating dendritic cells in LTAgvax treated tumors compared to pVax treated tumors (**Supplementary Figure 8F**). There was no difference in percentages of myeloid derived MDSCs (M-MDSCs) between both groups, LTAgvax treated tumors had significantly fewer PMN derived MDSCs (PMN-MDSCs, **Figure 6H**). We observed a significant decrease in the ratio of M-MDSC/CD8 T cells (**Figure 6I**) and PMN-MDSC/CD8 T cells (**Figure 6J**) in LTAgvax treated tumors suggesting that there are fewer immunosuppressive cells per CD8+ T cells. There were no changes in the anti-tumor M1 macrophages between both groups, but we saw a decrease in the pro-tumor M2 macrophage population in the LTAgvax treated tumors (**Figure 6K**). This translated into a significantly lower M2 macrophage/CD8 T cell ratio (**Figure 6L**) in LTAgvax treated tumors again highlighting that vaccinating with LTAgvax leads to fewer intratumoral immunosuppressive cells compared to cytotoxic CD8+ T cells.

Overall, flow cytometry analysis of tumors demonstrates that LTAgvax treatment significantly reshapes the TME leading to fewer PMN-MDSCs and M2 macrophages and an increased CD4+ and CD8+ T cell infiltration. The fewer immunosuppressive cells allow for enhanced activation of the CD8+ T cells which also retain the effector memory phenotype longer which allows them keep killing the tumor cells.

### Global population coverage of LTAgvax

**Figure 7:**
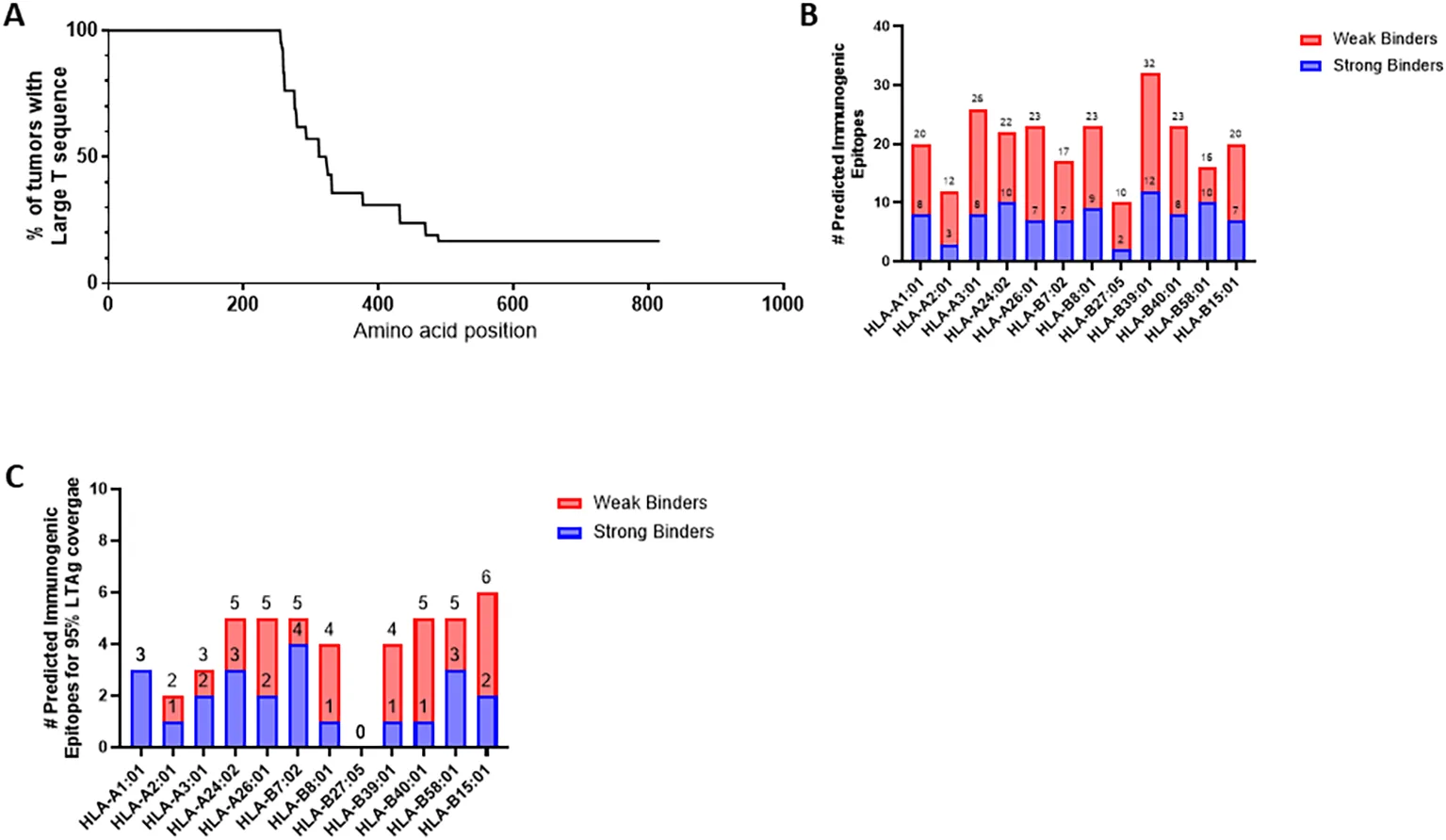
LTAgvax is predicted to be immunogenic in a global panel of human MHC. **(A)** Extent of LTAg truncation in human MCC samples. **(B)** Number of predicted immunogenic epitopes in LTAgvax across 12 different human class 1 MHC types. **(C)** Number of predicted immunogenic epitopes that would cover LTAg in 95% of MCPyV+ MCC tumors across 12 different human class 1 MHC types.

Several studies report LTAg truncation in MCC tumors. The truncated LTAg is reported to be more stable while retaining the ability to bind to Rb, inhibiting its activity and driving tumorigenesis (31–33). We studied the LTAg sequences of 42 available on Genbank to better understand the LTAg truncations. Based on our analysis, the 1^st^ 254 amino acids are present in 100% of the MCC tumors and 50% of the tumors containing up to 322 amino acids. Only ~17% of LTAg sequences we analyzed had the full length LTAg sequence (**Figure 7A**).

To better understand how the LTAgvax would work in humans, we used NetMHCpan (Version 4.0) to predict which LTAgvax epitopes would bind to human MHC alleles (34). We used 12 representative HLA supertypes which cover >90% of the global human population for these predictions. Our analysis found that for each Class I MHC allele, there are at least 10 distinct epitopes which are predicted to bind to the respective Class 1 MHC allele with high/medium affinity and would be either strong or weak binders (**Figure 7B**). If we restrict our analysis to the first 258 amino acids which would cover ~95% of the MCC tumors, we still get 2 or more distinct epitopes which would be immunogenic for 11/12 alleles tested (**Figure 7C**). These data support that LTAgvax would elicit CD8+ T cell responses in human population with high coverage of LTAg expression within MCC tumors.

## Discussion

Merkel cell carcinoma (MCC) remains a disease with high lethality and limited durable therapy options, especially for patients whose tumors either do not respond to or relapse after ICI therapy (1). MCPyV, which is associated with ~80% of MCC tumors presents an attractive target for therapeutic cancer vaccines due to its constitutive expression, expression limited to tumor cells and oncogenic dependence on viral T antigens (3, 15).

In this study, we demonstrate that synDNA vaccines encoding consensus MCPyV LTAg and STAg sequences induce robust, multifunctional immune responses in preclinical models. We observed strong cellular responses in C57Bl6 mice from CD4+ and CD8+ T cells. Both CD4+ and CD8+ T cells had polyfunctional subsets, simultaneously secreting IFNγ, TNFα and IL2. This T cell phenotype has been associated with better effector capacity, T cell persistence and anti-tumor capacity. The induced CD8+ T cells also simultaneously expressed the degranulation marker CD107a and Tbet highlight the killing potential of the vaccine induced T cells, which is essential for control of established malignancies.

Both vaccines elicited strong, polyfunctional cellular responses in outbred CD-1 mice supporting immunogenicity across more diverse mouse MHC types. The LTAgvax was effective in controlling growth of LTAg expressing B16 tumors in a minimal residual disease model including 50% of the mice that had a complete response. This enhanced tumor control also significantly improved the survival of tumor bearing mice. LTAgvax significantly remodeled the tumor microenvironment, making it more favorable for T cell infiltration and activation, leading to improved tumor killing in a therapeutic setting and improved survival of the tumor bearing mice. This was further enhanced when LTAgvax was combined with anti-PD1 therapy as 100% of the mice were tumor free at the end of the experiment, leading to further improvement in animal survival. Finally, we showed that across 12 different human MHC types covering >90% of the global human population, the LTAgvax had at least 10 distinct epitopes that were predicted to be immunogenic and 11/12 human MHCs had at least 2 epitopes derived from the first 254 amino acids of LTAg (covering ~95% of MCPyV+ MCC tumors) that were predicted to be immunogenic. These data highlight that LTAgvax could be immunogenic across the global human population and potentially be useful in controlling most of the MCPyV+ MCC tumors.

Other groups have developed vaccines targeting MCPyV for MCC therapy. Zeng et al. first demonstrated that a DNA vaccine targeting the N-terminal region of LTAg elicited antigen specific T cell responses and demonstrated tumor control in B16-LTAg mouse model. Interestingly, the anti-tumor activity was primary driven by CD4+ T cells (35). While CD4+ T cells can be important for tumor control, clinically, CD8+ T cells are the primarily responsible for effective tumor control (24). Accordingly, strategies that enhance the magnitude and quality of CD8+ T cell responses may further improve therapeutic efficacy. The same group extended this strategy to STAg, showing CD8+ T cell induction and tumor control in a B16-STAg mouse model (36). Both studies establish the value of targeting LTAg and STAg for MCC therapy. However, both studies used a single LTAg/STAg sequence instead of using a consensus sequence derived from multiple viral isolates (35, 36). This could limit the efficacy of these vaccines against global MCPyV strains limiting translational impact. Together, these considerations highlight opportunities for further optimization of MCPyV LTAg directed vaccine design to enhance translational potential.

More recently, Rosean et al. demonstrated that a lysosomal-targeting (LAMP1) fusion DNA vaccine encoding a detoxified form of LTAg enhanced CD4+ T cell responses, increased intratumoral T cell infiltration, and delay tumor B16-LTAg growth, while also demonstrating synergy with PD-1 checkpoint blockade (37). This platform has now advanced into first-in-human evaluation in MCC patients who have undergone standard of care and exhibit no evidence of active disease (NCT05422781), the results of which will be exciting to see. To the best of our knowledge, this is the only vaccine candidate in clinical testing targeting the LTAg to prevent MCC recurrence. Collectively, these studies highlight both the promise of MCPyV T antigen targeting vaccines and recurring translational challenges, specifically, the variable induction of CD8+ T cell responses in animal models and testing of these constructs in in-bred C57Bl6 animals only.

Our synthetic consensus LTAg and STAg synDNA vaccines are designed to address several limitations of earlier studies. We optimized antigenic sequence from several highly conserved viral sequences to broaden coverage across globally circulating MCPyV variants, which is important for enhancing utility of the vaccine. In contrast to truncated LTAg designs, our strategy incorporates full length consensus LTAg and STAg, allowing for targeting of 100% MCPyV+ tumors and providing additional immunogenic sequences for T cell targeting. Consistent with this design rationale, vaccination elicited polyfunctional CD4+ and CD8+ T cells producing IFNγ, TNFα, and IL-2, as well as CD8+ T cells expressing T-bet and secreting CD107a, indicative of cytotoxic effector differentiation. These responses were observed against peptide pools derived from the entire LTAg and STAg sequence highlighting that our vaccine contains multiple immunogenic regions. Our lab has previously developed synDNA vaccines targeting neoantigens using the same netMHC prediction algorithm as used in this manuscript to identify immunogenic epitopes from LTAgvax and STAgvax in C57BL6 mice (18, 24). This work was translated into a Phase 1 clinical trial for Hepatocellular carcinoma patients (Published in Nature Medicine, 2024) where the authors reported vaccine induced immune responses against 64% of the neoantigens targeted (30), which is the highest number reported in the cancer neoantigen field. A second clinical trial in human glioblastoma patients (published in Nature Cancer, 2026) demonstrated similarly high epitope responses (38). These data establish the translational link between *in silico* predicted immunogenic epitopes in mice in our studies as we have published and clinical immune responses observed in human patients. The induction of immune responses in outbred mice suggests that this platform may retain immunogenicity across diverse genetic backgrounds, a key translational requirement for human deployment. We observed robust control of LTAg+ tumors with LTAgvax in a minimal residual disease model. Finally, in a therapeutic challenge, LTAgvax demonstrated significant control of B16-LTAg tumors which was further improved in combination with anti-PD1 therapy including 100% of the mice that received combination therapy being tumor free. Mechanistically, LTAgvax created a more favorable tumor microenvironment for the CD8+ T cells with fewer immunosuppressive cells and greater CD8+ T cells. This allowed the T cells to be more active and stay for longer in the tumors explaining the improved tumor control observed. It has been reported that ~52% of MCC patients develop antibodies to MCPyV oncoproteins and that the seropositive patients had a 42% lower rate of recurrence compared to seronegative patients highlighting the importance of humoral immune responses in MCC disease recurrence (39). We observed strong antibody responses against both LTAg and STAg in response to the respective vaccines. Collectively, these data suggest that in a clinical setting, LTAgvax and STAgvax could be useful in preventing or delaying tumor recurrence and positively impacting the survival rates for MCC patients.

The B16 transduced LTAg cells used in our study provide several advantages. Viral antigens are foreign proteins and not subject to central or peripheral tolerance in mice, hence the magnitude of vaccine induced immune responses would not be impacted. However, these cells also present certain limitations. B16-LTAg cells do not depend on LTAg expression for oncogenesis and could lose or downregulate antigen expression without any cost. This would downplay the vaccine induced tumor control observed in our study. In human MCPyV tumors, LTAg is a major driver of oncogenesis and tumors would not be able to downregulate its expression. B16 cells have also lower expression of MHC 1 pathway components including TAP (peptide transporter), LMP2, LMP7 and LMP10 (proteasome subunits) and chaperone protein tapasin (40). This would further attenuate the vaccine induced CD8+ T cells’ ability to control tumor. Future studies by transducing LTAg into alternate cell lines that do not downregulate MHC 1 pathway components could address some of these caveats.

In conclusion, synDNA encoding LTAg and STAg targeting vaccines are immunogenic in inbred and outbred mice, generate polyfunctional CD4+ and CD8+ T cells and effectively control growth of LTAg expressing tumors. As relates to the human population, across 12 distinct HLA types, covering >90% of the global population, there are at least 10 LTAg derived epitopes that are predicted to be immunogenic. This study highlights that synDNA vaccines against LTAg and STAg could be important for immunotherapy of MCC tumors.

## Materials and methods

### Animals and cell lines

6–8 week old female C57BL6 or CD1 mice were purchased from Jackson Laboratory. All animal experiments were approved by the Wistar Institute Institutional Animal Care and Use Committee. B16F10 cells were purchased from ATCC and maintained at low passage (<10) in D10 media. We generated B16-LTAg cells using lentiviral transduction as previously described (23). The cell lines were regularly tested for mycoplasma. Mice were immunized with 25 μg of each plasmid by intramuscular injection into the tibialis anterior (TA) muscle, followed by electroporation using the CELLECTRA-3P device (Inovio Pharmaceuticals). Two 0.1-amp constant current square-wave pulses were delivered through a triangular three-electrode array. For immunogenicity studies and tumor challenge studies, mice were given a total of three immunizations at 2-week intervals.

### Vaccine design

LTAgvax and STAgvax were designed using consensus sequences from all available sequences in NCBI database. The consensus sequence was obtained by aligning these sequences in Clustal W. Additional mutations were added to both sequences to abolish oncogenic activity. To elaborate, *D44N- blocks binding to chaperone proteins; *W209A- blocks binding to hVam6p; *E216K- blocks binding to Rb and prevents transformation; *L142A- blocks binding to PP2A; *91-95LKDYM->AAAAA- blocks binding to Fbxw7 and prevents transformation (41). The sequences were RNA and codon optimized with an IgE leader sequence and kozak sequence added at the N terminus. This was cloned into a modified high expression pVax1 vector. The amino acid sequences are also shown in **Supplementary Figure 9**.

### IFNγ ELISPOT

6–8 week old C57BL6 mice were vaccinated three times at 2-week intervals. 1 week following the final vaccination, splenocytes were harvested and co-incubated with each LTAg or STAg derived peptide pools comprising 15-mer peptides overlapping by 9 aa (5 μg/mL). Splenocytes were cultured at 37 °C. 24 h later, we performed the murine IFNγ ELISPOT using MABTECH Mouse IFNγ ELISpot^PLUS^ plates according to the manufacturer’s instructions. Concavalin A was used as a positive control.

### Intracellular cytokine staining and flow cytometry

2 million splenocytes per mouse were stimulated with LTAg/STAg derived peptides for 5 hr in the presence of GolgiStop GolgiPlug (BD Bioscience). Phorbol myristate acetate (PMA, BD Bioscience) and complete media were used as positive and negative controls, respectively. Cells were incubated with FITC α-mouse CD107a (clone 1D4B, Biolegend) during the stimulation to stain for degranulation. After stimulation, cells were washed and stained for viability using LIVE/DEAD violet. Following surface staining, cells were fixed and permeabilized using a FoxP3/transcription factor fixation/permeabilization kit (eBioscience). The following antibodies were used in these studies: PECy5 αCD3 (clone 145-2C11, BD PharMingen), BV510 αCD4 (clone RM4-5, Biolegend), BV605 αTNF-α (clone MP6-XT22), PE αT-bet (clone 4B10, Biolegend), APCCy7 αCD8 (clone 53-6.7, Biolegend) and APC αIFNγ (clone XMG1.2, Biolegend). Data was acquired on a BD FACSymphony cytometer (BD Bioscience) and analyzed using FlowJo.

### Minimal residual disease tumor challenge

6–8 week old female C57BL6 mice were vaccinated with indicated construct 3x at 2-week intervals. One week after the final dose, 200,000 B16-LTAg cells (in PBS) were injected on the right flank subcutaneously. Tumor size was monitored via caliper measurements. Mice were euthanized when the length of tumor reached 20 mm or tumor volume reached greater than 2,000 mm3. Tumor volume was calculated using the formula V = [(length × width2) × 3.14]/2, where width is considered the side with smaller measurement. Graph represents data from two independent experiments (n=6 mice/group).

### Therapeutic tumor challenge

100,000 B16-LTAg cells (in PBS) were injected subcutaneously on the right flank of 6–8 week old C57BL6 mice. 7 days post tumor challenge, we initiated treatment with either LTAgvax (25ug) or anti-PD1 antibody (Bioxcell, 100ug/dose) or both. LTAgvax was administered every 7 days for a total of 3 doses. Anti-PD1 antibody was administered bi-weekly for a total of 6 doses. Mice were euthanized when the length of tumor reached 20 mm or tumor volume reached greater than 2,000 mm3. Tumor volume was calculated using the formula V = [(length × width2) × 3.14]/2, where width is considered the side with smaller measurement. (n=5 mice/group).

Three tumors from pVax and LTAgvax treated groups were analyzed for immune cell infiltrate at the terminal timepoint. The tumors were broken down into single cell suspensions using the mouse tumor dissociated kit (Miltenyi Biotec) as per the manufacturer’s instructions. Cells were then stained for Live/Dead (Zombi Yellow), CD45 (PerCp-Cy5.5), CD8 (BV510), CD4 (BV421), PD1 (FITC), CD69 (APC), CD44 (AF700), CD62L(BV711), CD86 (PE-Cy7) for TIL analysis. For Myeloid cells we used Live/Dead (Zombi Yellow), CD45 (PerCp-Cy5.5), CD11b (BV650), F4/80 (BV421), CD206 (FITC), CD86 (PE-Cy7), CD11c (APC), Ly6C (BV785) and Ly6G (APC-Cy7). Data was acquired on a BD FACSymphony cytometer (BD Bioscience) and analyzed using FlowJo.

### Statistical analysis

All statistical analyses were performed using the latest version of GraphPad Prism. For each experiment, error bars represent the mean ± the SEM. For all experiments, statistical significance was determined by one- or two-way ANOVA while significance for mouse survival was determined by Gehan-Breslow-Wilcoxon test.

## Data Availability

The original contributions presented in the study are included in the article/**Supplementary Material**. Further inquiries can be directed to the corresponding author.
