# Supplementary material for: Synthetic consensus DNA vaccines against Merkel cell polyomavirus large and small T antigens induce robust polyfunctional T cell responses
Source: Front Immunol. 2026 Sep 4;17:1840943. doi: 10.3389/fimmu.2026.1840943 (PMC13585920; doi:10.3389/fimmu.2026.1840943)
Supplement: Supplementary file 1 [file DataSheet1.pdf]

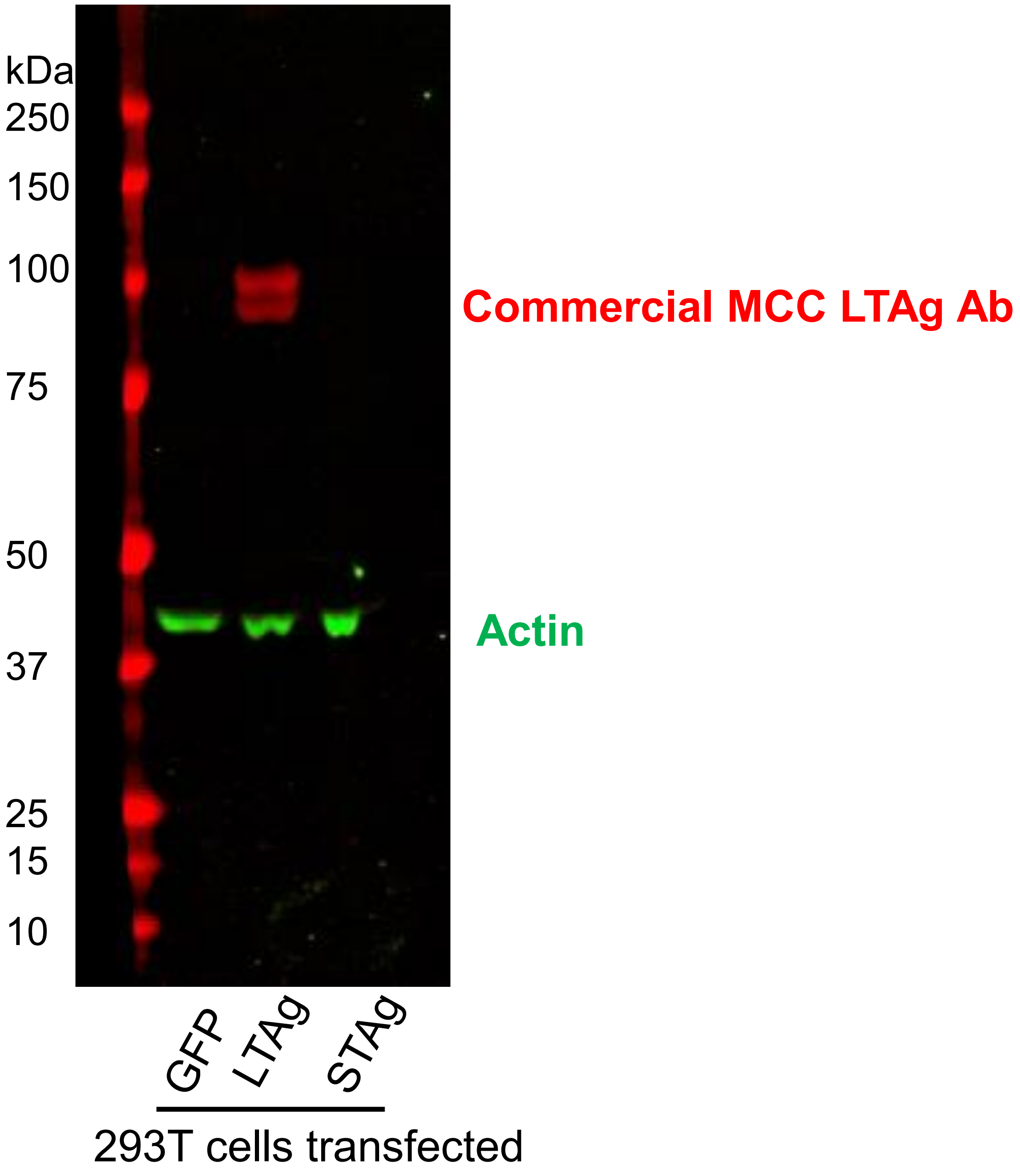

Supplementary Figure 1: *in vitro* expression of LTAgvax in 293T cells

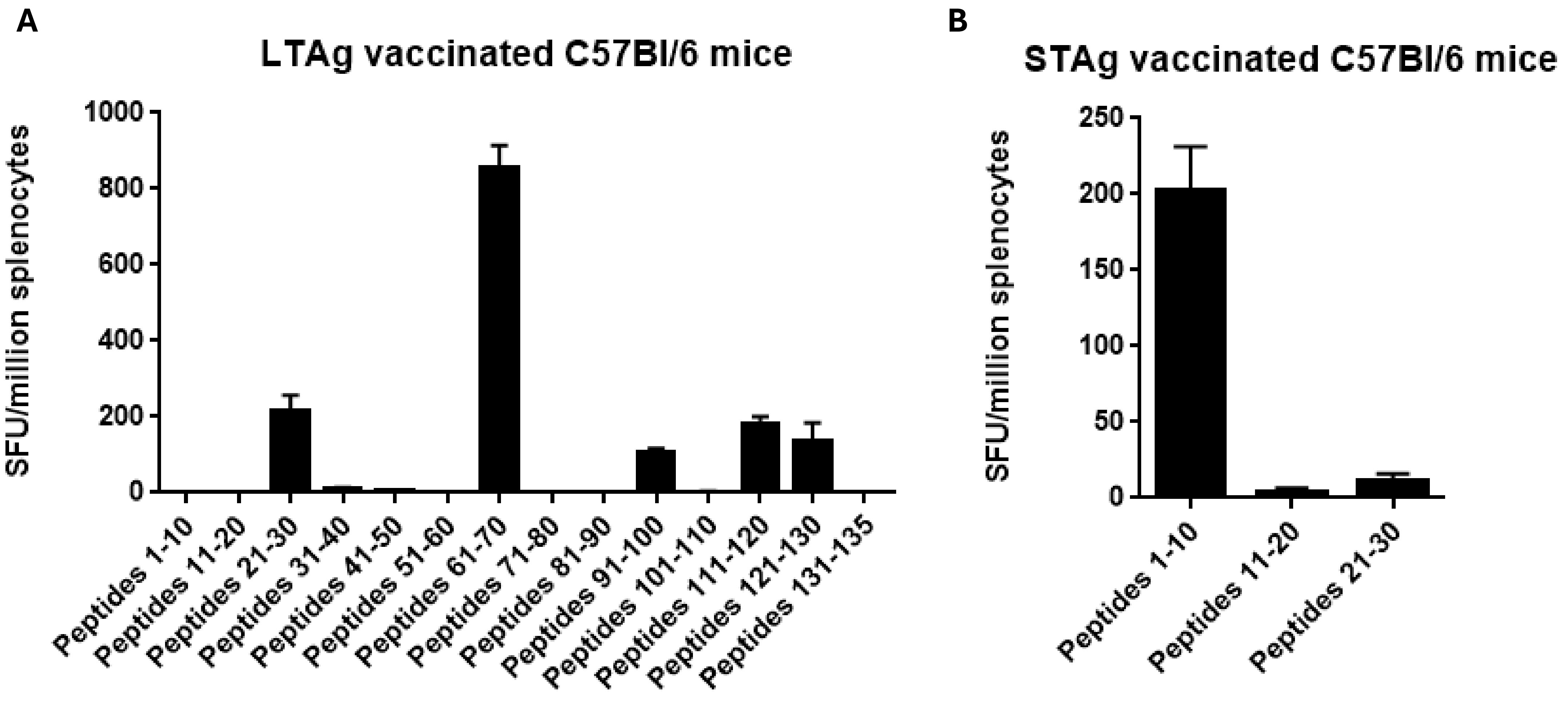

Supplementary Figure 2: Mapping of immunodominant epitopes in C57Bl/6 mice

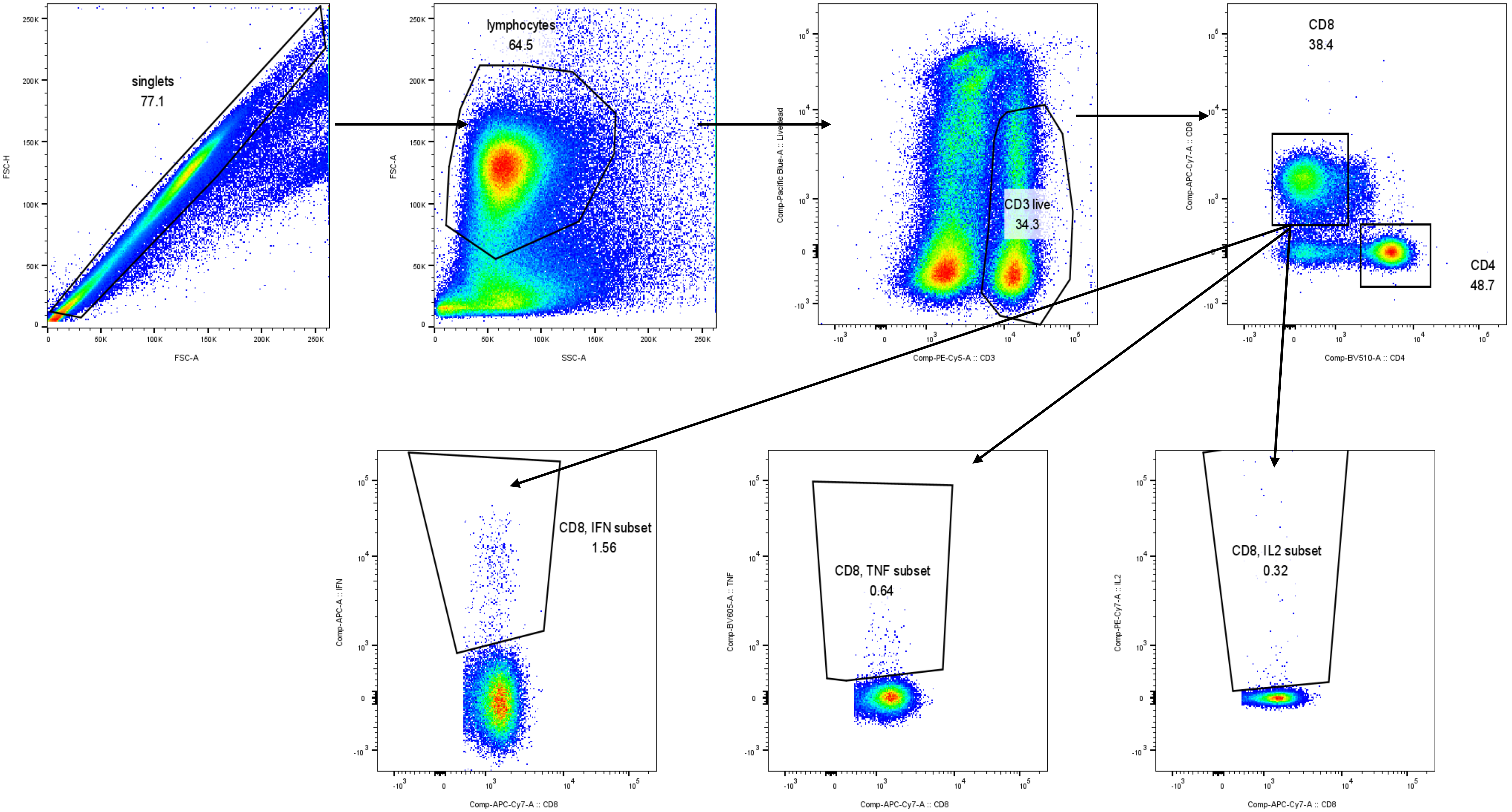

Supplementary Figure 3: Gating strategy for Figure 2 D-F

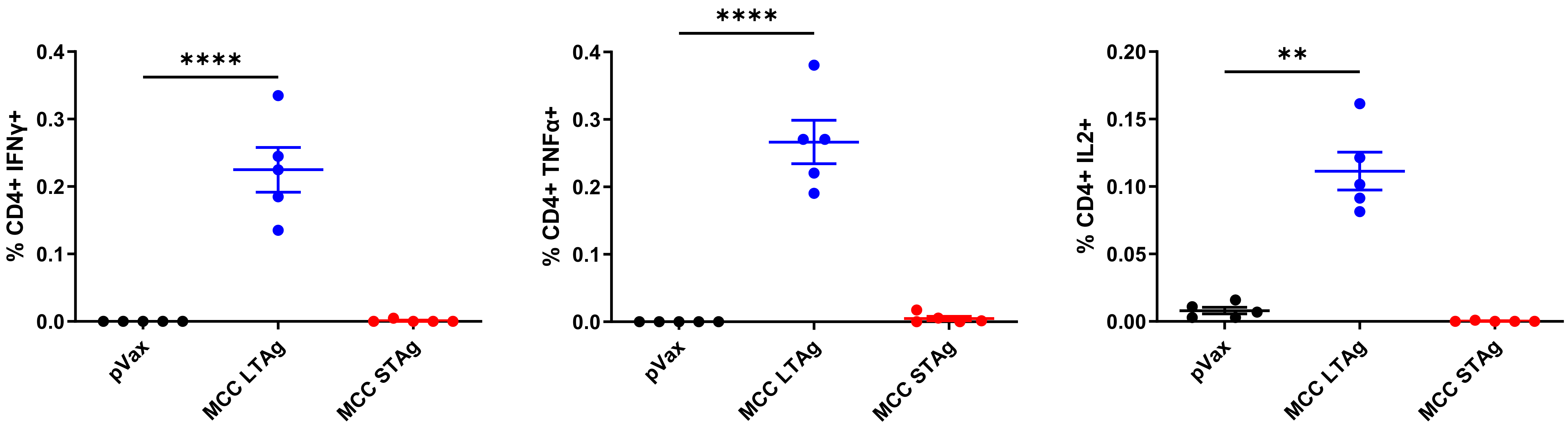

Supplementary Figure 4: CD4+ T cell response elicited by LTAgvax in C57bl6 mice

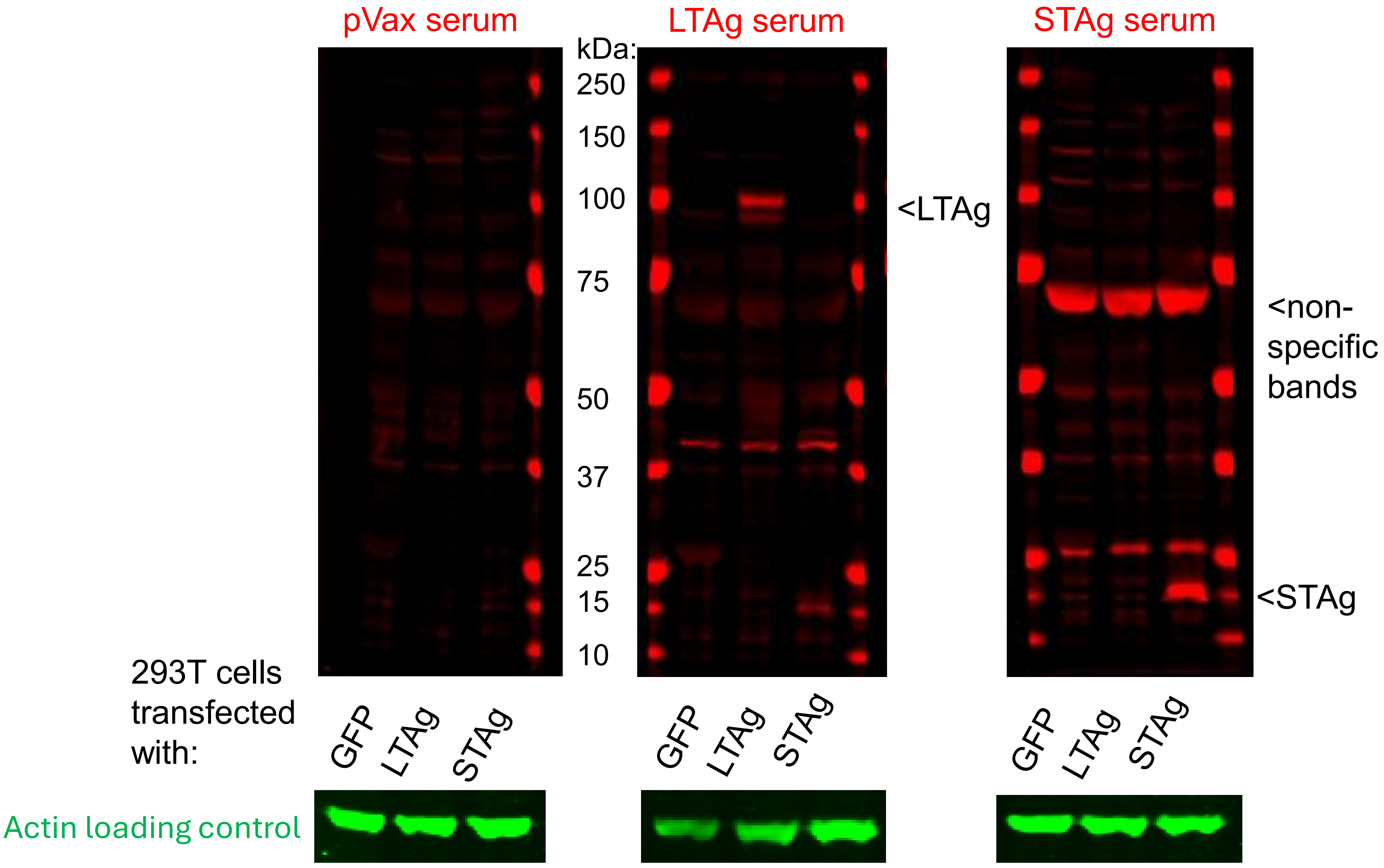

Supplementary Figure 5: Large T and Small T antigen vaccines generate humoral responses

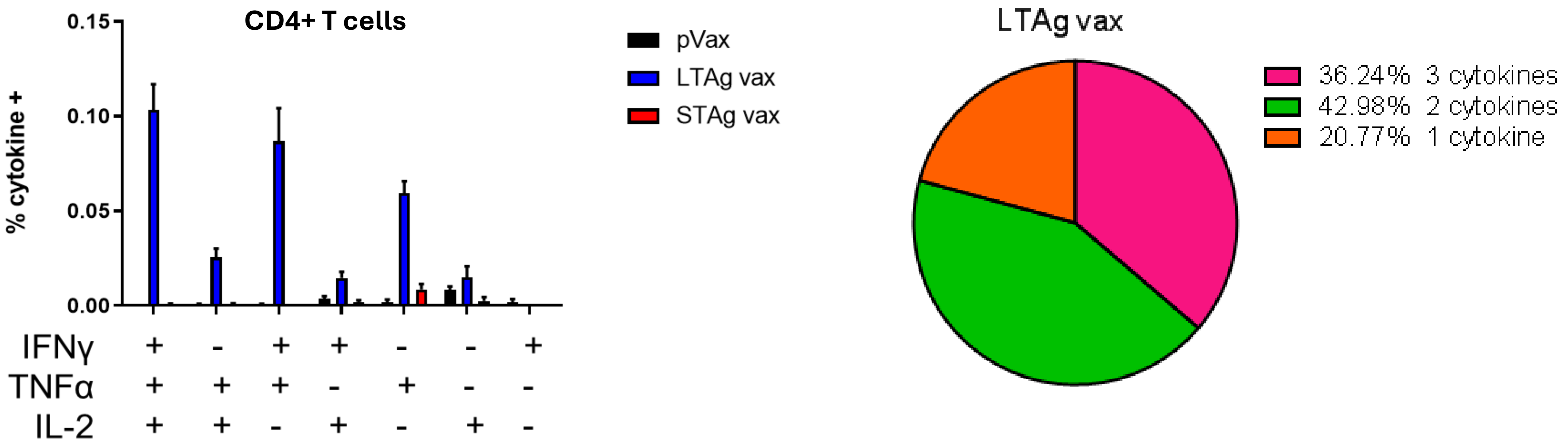

Supplementary Figure 6: Triple, double and single positive CD4+ T cells from LTAgvax and STAgvax.

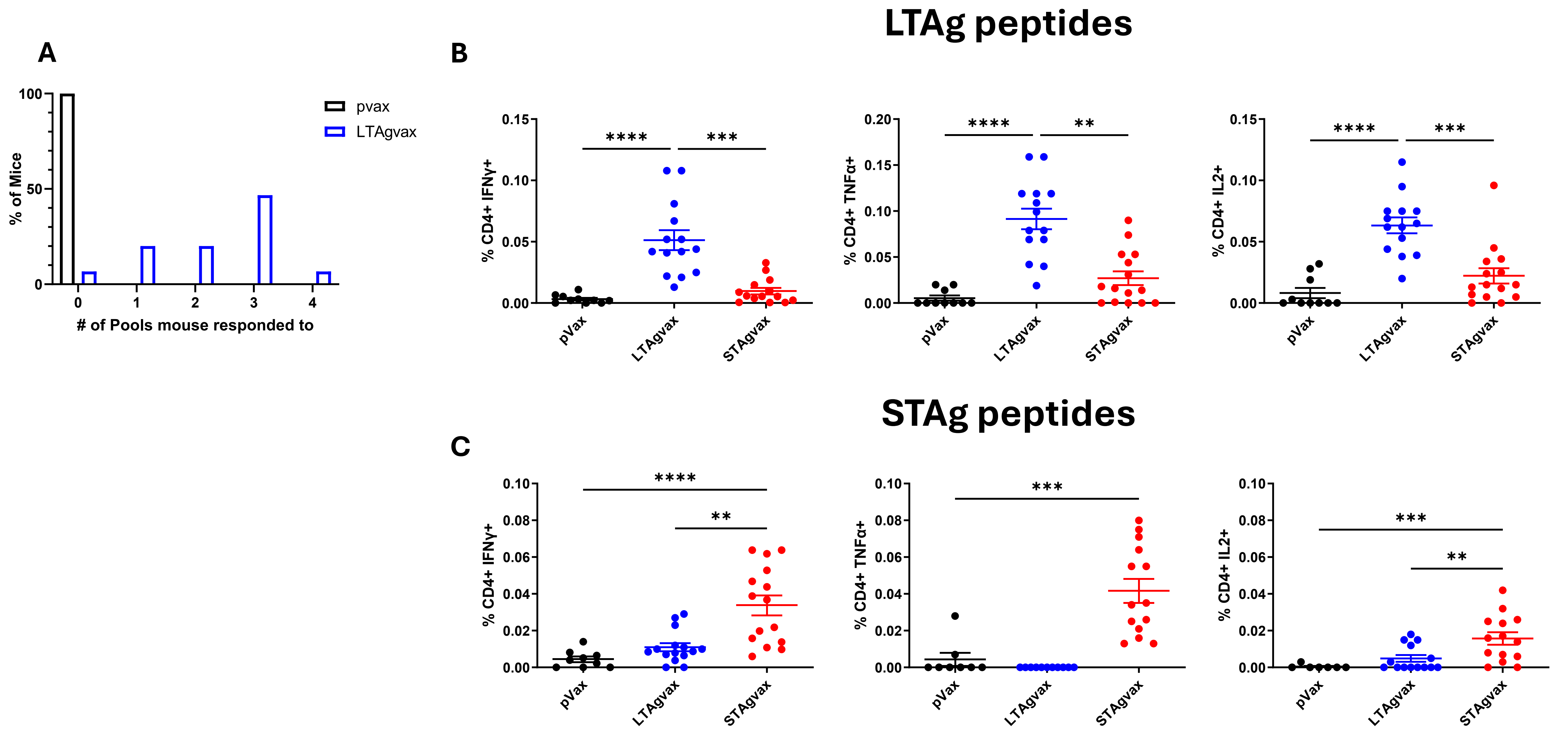

Supplementary Figure 7: A) Percentage of CD-1 mice that responded to 0,1, 2,3 or 4 pools after immunization with LTAgvax. B and C) CD4+ T cell response in CD-1 mice from LTAgvax or STAgvax when stimulated with peptides derived from LTAg or STAg

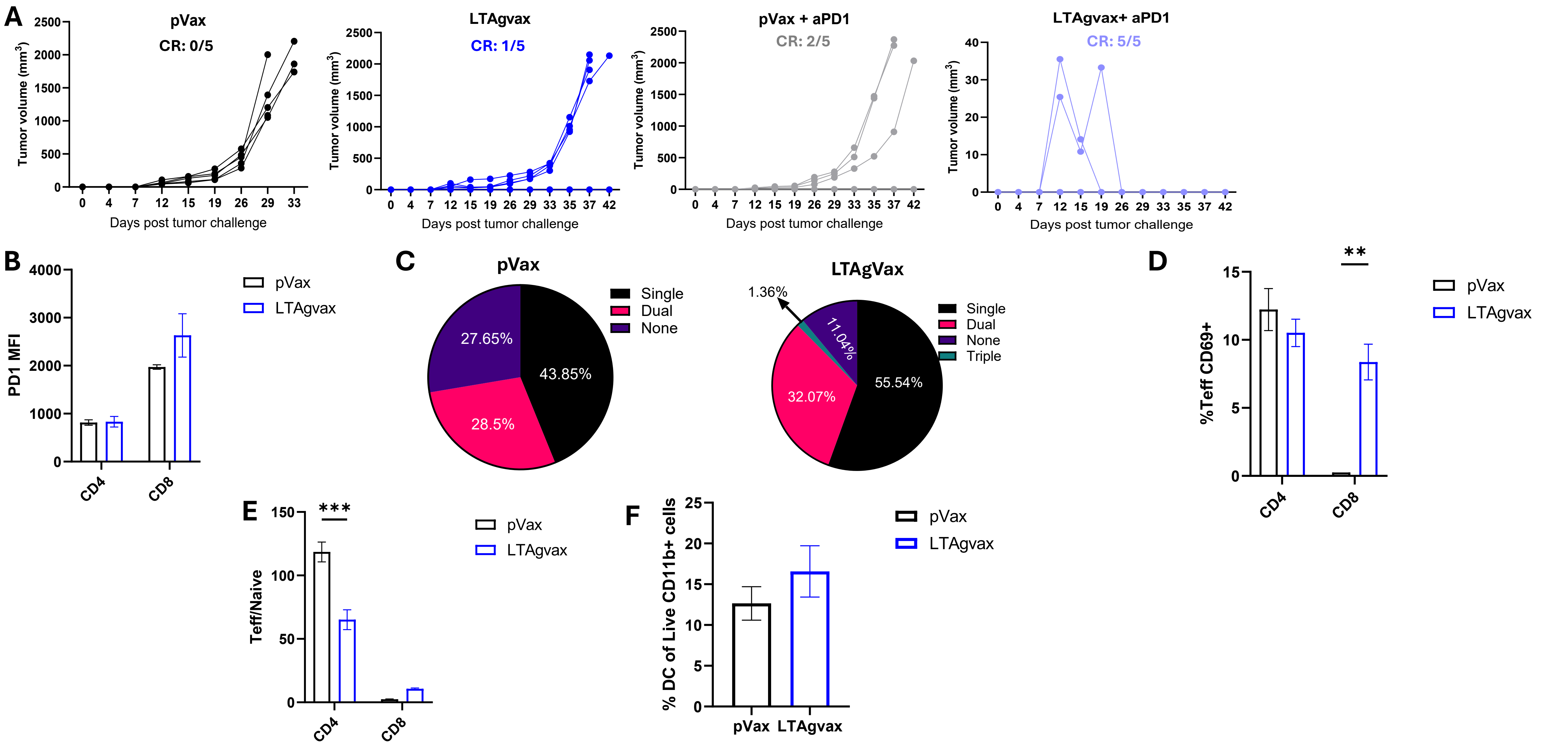

Supplementary Figure 8: A) Individual B16-LTAg tumor sizes in mice treated with pVax, LTAgvax, anti-PD1 or LTAgvax + anti-PD1. B)PD1 MFI on CD4 and CD8 T cells in tumors of mice treated with pvax or LTAgvax. C) Ratio of Teff/Naïve CD4 and CD8 T cells in tumors of mice treated with pVax or LTAgvax. D) % CD69+ Teff CD4 and CD8 T cells in tumors of mice treated with pVax or LTAgvax. E) % Dendritic cells in tumors of mice treated with pVax or LTAgvax. F) % CD8+ T cells expressing zero, one, two or three activation markers in tumors of mice treated with pVax or LTAgvax.

MCPyV STAgvax:

MDWTWILFLVAAATRVHSMDLVLNRKEREALCKLLEIAPNCYGNIPLMKAAFKRSCLKHHPNKGGNPVIMMELNTLWSKFQQNIHKLRSDFSMFDEVSTKFPWE
EYGTAAAAAQSGYNARFCRGPGCMLKQLRDSKCACISCKLSRQHCSLKTLKQKNCATWGECFCYQCFILWFGFPPTWESFDWWQKTLEETDYCLLHLHLF

MCPyV LTAgvax:

MDWTWILFLVAAATRVHSMDLVLNRKEREALCKLLEIAPNCYGNIPLMKAAFKRSCLKHHPNKGGNPVIMMELNTLWSKFQQNIHKLRSDFSMFDEVDEAPIYG
TTKFKEWWRSGGFSFGKAYEYGPNPHGTNSRSRKPSSNASRGAPSGSSPPHSQSSSSGYGSFSASQASDSQSRGPDIPPEHHEEPTSSSGSSSREETTNSGRE
SSTPNGTSVPRNSSRTDGTAEDLFCDKSLSSPEPPSSSEEPEEPPSSRSSPRQPPSSSAEEASSSQFTDEEYRSSSFTTPKTPPPFSRKRKFGGSRSSASSASSASFT
STPPKPKKNRETPVPTDFPIDLSDYLSHAVYSNKTVSCFAIYTTSDKAIELYDKIEKFKVDFKSRHACELGCILLFITLSKHRVSAIKNFCSTFCTISFLICKGVNKMPEM
YNNLCKPPYKLLQENKPLLNYEFQEKEKEASCNWNLVAEFACEYELDDHFIILAHYLDFAKPFPCQKCENRSRLKPHKAHEAHHSNAKLFYESKSQKTICQQAAD
TVLAKRRLEMLEMTRTEMLCKKFKKHLERLRDLDTIDLLYYMGGVAWYCCLFEEFEKKLQKIIQLLTENIPKYRNIWFKGPINSGKTSFAAALIDLLEGKALNINCPSD
KLPFELGCALDKFMVVFEDVKGQNSLNKDLQPGQGINNLDNLRDHLDGAVAVSLEKKHVNKKHQIFPPCIVTANDYFIPKTLIARFSYTLHFSPKANLRDSLDQN
MEIRKRRILQSGTTLLLCLIWCLPDTTFKPCLQEEIKNWKQILQSEISYGKFCQMIENVEAGQDPLLNILIEEEGPEETEETQDSGTFSQ**

Supplementary Figure 9: Syncon sequences for MCPyV STAgvax and LTAgvax.
